# Supplementary material for: A Meta-Analysis of the Effectiveness of Telemedicine in Glycemic Management among Patients with Type 2 Diabetes in Primary Care
Source: Int J Environ Res Public Health. 2022 Mar 31;19(7):4173. doi: 10.3390/ijerph19074173 (PMC8999008; doi:10.3390/ijerph19074173)
Supplement: Supplementary file 1 [file ijerph-19-04173-s001.zip › ijerph-1641010-supplementary.pdf]

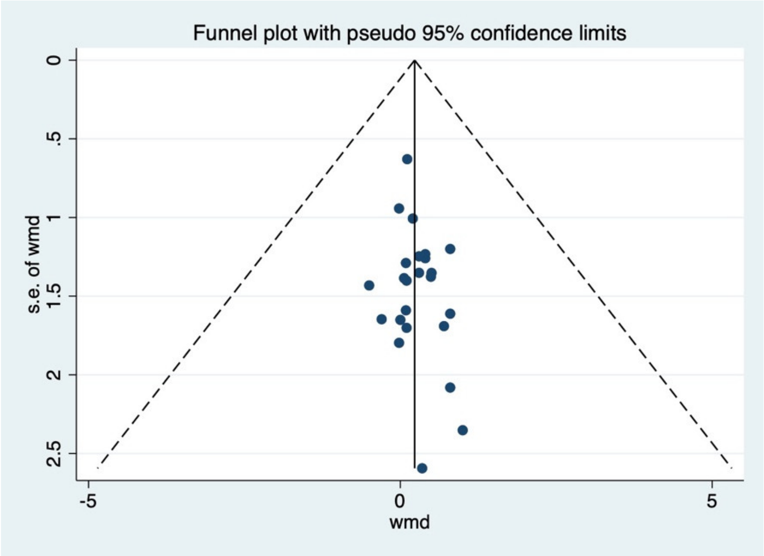

Figure S1. Funnel plot evaluating publication bias with regard to HbA1c change for all studies.

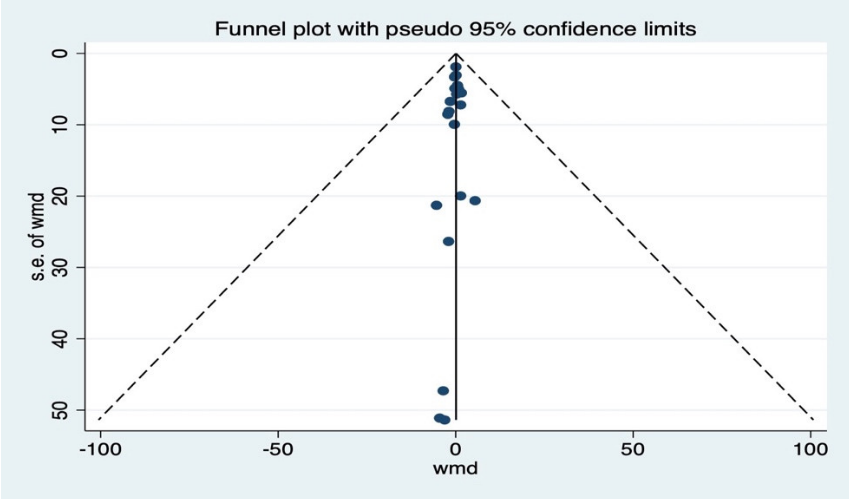

Figure S2. Funnel plot evaluating publication bias with regard to wieght change for all studies.

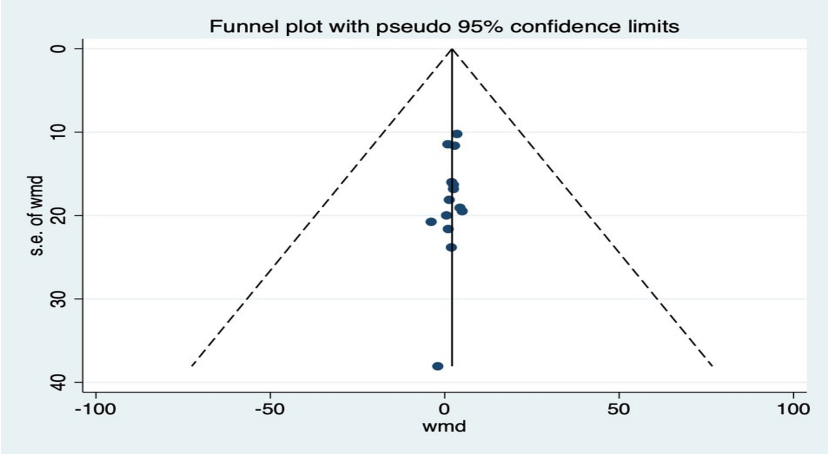

Figure S3. Funnel plot evaluating publication bias with regard to SBP change for all studies.

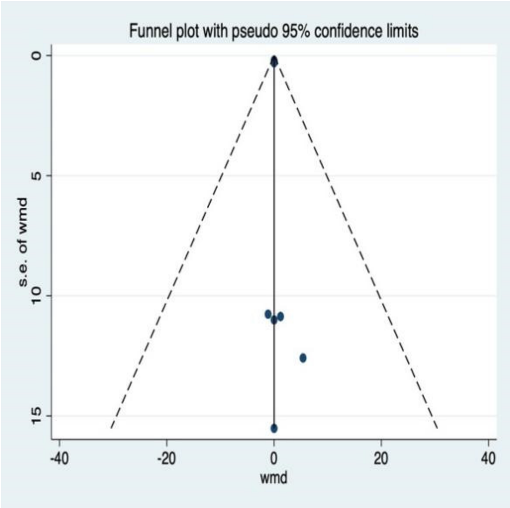

Figure S4. Funnel plot evaluating publication bias with regard to HDL change for all studies.

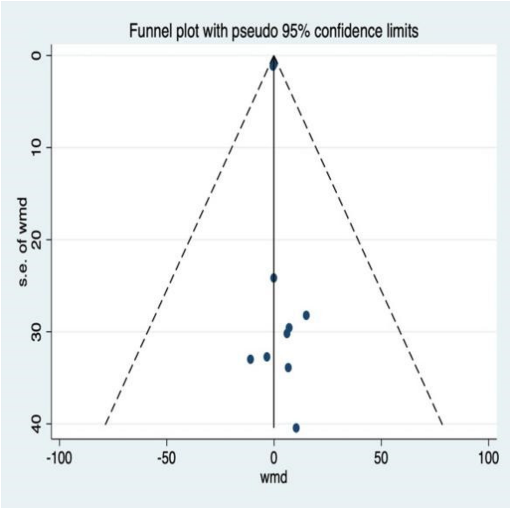

Figure S5. Funnel plot evaluating publication bias with regard to LDL change for all studies.
